# Use of Fish Scale-Derived BioCornea to Seal Full-Thickness Corneal Perforations in Pig Models

**DOI:** 10.1371/journal.pone.0143511

**Published:** 2015-11-24

**Authors:** Shih-Cheng Chen, Niklas Telinius, Han-Tse Lin, Min-Chang Huang, Chien-Chen Lin, Cheng-Hung Chou, Jesper Hjortdal

**Affiliations:** 1 Aeon Astron Europe B.V., Leiden, The Netherlands; 2 Department of Ophthalmology, Aarhus University Hospital, Aarhus, Denmark; 3 Body Organ Biomedical Corp., Taipei, Taiwan; University of Reading, UNITED KINGDOM

## Abstract

The aim of this study was to test the use of BioCornea, a fish scale-derived collagen matrix for sealing full-thickness corneal perforations in mini-pigs.

Two series of experiments were carried out in 8 Lan-Yu and 3 Göttingen mini-pigs, respectively. A 2mm central full thickness corneal perforation was made with surgical scissors and 2mm trephines. The perforations were sealed immediately by suturing BioCornea to the wounded cornea. The conditions of each patched cornea were followed-up daily for 3 or 4 days. Status of operated eyes was assessed with slit lamp examination or optical coherence tomography (OCT). Animals were sacrificed after the study period and the corneas operated were fixated for histological examination.

Both OCT imaging and handheld slit lamp observations indicated that a stable ocular integrity of the perforated corneas was maintained, showing no leakage of aqueous humor, normal depth of anterior chamber and only mild swelling of the wounded cornea. Hematoxylin and eosin staining of the patched cornea showed no epithelial ingrowths to the perforated wounds and no severe leucocyte infiltration of the stroma.

The fish scale-derived BioCornea is capable to seal full-thickness corneal perforation and stabilize the integrity of ocular anterior chamber in pre-clinic mini-pig models. BioCornea seems to be a safe and effective alternative for emergency treatment of corneal perforations.

## Introduction

Corneal perforation is a serious condition that may result in devastating visual consequences. Early and late complications to corneal perforations include cataract, glaucoma, endophthalmitis, and ultimately the loss of the eye [1]. Infection, inflammation, and trauma are the leading causes of corneal perforations though other causes such as exposure or degeneration are also reported [2–6]. Primary treatment is to close the perforation and restore the integrity of the globe. Early diagnosis and proper emergency management can promote better visual recovery [7–9]. Large perforations usually require urgent surgical and non-surgical treatments to close the wound and restore the integrity of the globe in order to prevent severe complications [3, 8, 9].

To date, surgical treatment of corneal perforations are broadly reported using amniotic membrane, cyanoacrylate-derivative glue, and fibrin glue with proper post-surgical medications [10–16]. Fibrin and cyanoacrylate derivative glue are the most used agents to treat corneal perforations. Nevertheless, good surgical outcome may rely on proper gluing techniques particularly in large full-thickness perforation [17–19].

Rijneveld and co-workers from Amsterdam Cornea Bank reported that a selected cornea donor pool, of which the donor corneas pass safety tests but fail to meet all of the other criteria for penetrating keratoplasty, can be preserved and used particularly for emergency corneal grafting [20–21]. However, most other regions in the world do not have banking facilities for emergency corneal grafting. Thus, a ready-to-use in-house device that can be applied to close and stabilize corneal injuries would be useful for emergency management of full-thickness corneal perforation.

Previously, the fish scale-derived collagen matrix, BioCornea, has been reported to show good biocompatibility when implanted in mice, rats, and rabbits, respectively [22–24]. In this pre-clinical study, we tested the safety and efficacy of applying BioCornea to seal full-thickness corneal perforations in Lan-yu and Göttingen minipigs.

## Material and Methods

### BioCornea

BioCornea is a premarket prototype developed by Aeon Astron Europe B.V. (Leiden, The Netherlands) and manufactured by Body Organ Biomedical Corp. (Taipei, Taiwan). It is a collagen matrix derived from tilapia fish scales by decellularization and decalcification [22]. In this study, the preparation procedure of BioCornea was adapted from the methods described by Lin et al. [22], followed by softening and thinning processes. In brief, fish-scales were selectively picked from the fresh skin of tilapia fishes. The decellularization process was done by sequential treatments of soaking the fish scales in sodium hydroxide (NaOH), *phosphate-buffered* saline (PBS), sodium chloride (NaCl) solutions, respectively. For decalcification ethylenediaminetetraacetic acid solution (EDTA) was used.

Subsequently, the decellularized and decalcified fish scales were trimmed with a laser punch to achieve a perfect round shape of a pre-specified diameter. Trimmed fish scales were then molded in the presence of 1, 4-butanediol diglycidyl ether (*BDDGE*) for a suitable curvature. The end product (BioCornea) was a transparent contact lens-like matrix predominantly consisting of type-I collagen [22].

On each BioCornea, suture holes were made with laser-drilling without affecting the smoothness of the surface. These holes were arranged in 2 rings from the center of BioCornea, of which 16 (type A) or 12 holes (type B) in total were made. BioCornea was sterilized with gamma-radiation prior to implementation in the animal. Two specifications of BioCornea were tested in this study as listed in Table 1.

**Table 1:** Specifications of the fish scale-derived corneal patches used in this study.

BioCornea specifications		
Type of BioCornea	A	B
Number of suture holes	16	12
Center thickness	450±50μm	200±50μm
Curvature	8.5±10%	8.5±10%
Diameter of the patch	8.5mm	6mm
Diameter of the suture holes	0.3mm	0.3mm
Distance from inner holes to the center	3±0.2μm	3±0.2μm
Distance from outer holes to the skirt	1±0.2μm	1±0.2μm

### Study animals

Eight 10 month-old Lan-yu Miniature Pigs (*Sus barbatus sumatranus*) weighing 50–55 kg and three 14 month-old Göttingen Minipigs (*Sus scrofa domestica*) weighing 24–35 kg were used in two series of experiments, respectively. Studied animals were anesthetized and incubated prior to the operations and kept anesthetized with propofol (14–20mg/kg/hour) and fentanyl (30–100μg/kg/hour) during the surgery. The animals were reared, operated, maintained, and sacrificed following the local regulations (Denmark and Taiwan) of animal studies. The Lan-yu minipig study protocol was approved by the Institutional Animal Care and Use Committee of Pigmodel Animal Technology Co., ltd, Taiwan (approval number PIG-103007). The Göttingen minipig study protocol was approved by the Danish Animal Experiments Inspectorate (approval number 2014-15-0201-00169). The studies adhered to the ARVO Statement for the Use of Animals in Ophthalmic and Vision Research. Prior to surgery, the conditions of the corneas to be operated were examined and confirmed as normal and healthy.

### Surgical procedures of Type-A BioCornea in Lan-Yu minipigs

The face of Lan-Yu minipig was sterilized with iodine solutions and the eye to be operated was lifted with 5–0 sutures through the upper and lower sclera prior to pre-surgical examination. Optical coherence tomography (OCT) was used to exam the condition of the corneas before and after the surgeries. Full-thickness corneal perforations were made with a 2-mm trephine and surgical scissors t the center of corneas. After the perforation was made, Healon was injected to the anterior chamber to support the cornea and maintain the anterior chamber angle.

The Type-A BioCornea was trimmed with an 8.5 mm-corneal punch prior to be used to seal the perforation. Carefully applying to cover the perforated site, the trimmed BioCornea was tightly sutured with 16 10–0 nylon single running suture through all the pre-made suture holes. After the surgery, proper antibiotic and steroids (prednisolone 1%; Pred Forte^®^, levofloxacin 0.5%; Cravit^®^ ophthalmic solution, and bethametason 0.1%; Rinderon^®^-A Ointment) were administered.

### Surgical procedures of Type-B BioCornea in Göttingen minipigs

Iodine (1mg/ml) and oxybuprokain (8mg/ml) were topically administrated to the eye, followed by injection of Viscoat (Alcon, Rødovre, Denmark) to the anterior chambers. A 2-mm in diameter full-thickness corneal perforation was made at the center of the eye of the mini-pig with a trephine. Subsequently, the corneal patch was used to seal the trephined corneal perforation. To patch the perforation, the center of the corneal patch was aligned to the center of the trephined wound to cover the perforation, followed by suturing with 12 nylon sutures (10–0) through the laser-made suture holes on the 12-hole patches. The sutures were made in order of rotational symmetry to keep the seal tight and balanced. After all the sutures were done, cefuroxim (1mg) was administered by intra-cameral injection, and a bandage contact lens was put in place to cover the sutured corneal patch.

### Post-operative observations

The pigs were sedated daily with dormicum (0.1–0.5mg/kg) to examine their post-surgical conditions. Occasionally stresnil (4mg/kg) was given depending on the condition of sedated mini-pigs. Optical coherence tomography (OCT) and handheld slit lamps were used to follow the conditions of perforated corneas and the anterior chambers of the Lan-Yu mini-pigs and the Göttingen mini-pigs, respectively. Topical chloramphenicol (0.5%) or prednisolone (1%), bethametason (0.1%) and levofloxacin (0.5%) was administered after each daily examination to the Göttingen and the Lan-Yu minipigs, respectively. Animal welfare was assessed daily in all pigs, and the Lan-Yu and the Göttingen mini-pigs were sacrificed with pentobarbital on day 3 and 4 post surgery, respectively. The operated corneas were harvested and fixated in formalin after sacrifice, followed by sectioning and HE staining according to standard procedure.

### Handheld slit lamp examination

Göttingen pigs were evaluated by daily inspection using a hand-held slit lamp. The following parameters were assessed: Secretion (0 (no) -4 (extensive)), Hyperemia (0 (no) -4(extensive)), Contact lens in place (Y/N), Corneal edema (0 (no) -4 (extensive)), Corneal infiltrate (Y/N), Biocornea in place (Y/N), Leakage (Y/N), Anterior chamber depth (Normal/decreased), Anterior chamber flare (Y/N), Iris hyperemia (0 (no) -4 (extensive)), Pupil centered (Y/N).

### Optical coherence tomography

The faces of Lan-Yu mini-pigs were sterilized with iodine solution, and Alcaine (0.5%) was applied topically on the eye. An eyelid speculum was applied to uncover the cornea and the eye was lifted with 1–2 nylon sutures (5/0) on conjunctiva. The OCT objective was situated in the front of cornea, and the distance and position between the objective and the cornea was adjusted accordingly to get clear images of central and peripheral cornea. The thickness of central cornea and the angle of anterior chamber were automatically calculated by the built-in module of the OCT machine (iVue SD-OCT, Optovue, USA).

### Statistics analysis

Corneal thickness and anterior chamber angle were analyzed using a one-way ANOVA and post Bonferroni test with significance set to p<0.05.

## Results

In this study we tested the efficacy and the safety of fish scale-derived BioCornea for sealing full-thickness corneal perforations. Two different specifications of BioCornea, the type A and B BioCorneas (Table 1) were tested.

**Fig 1:**
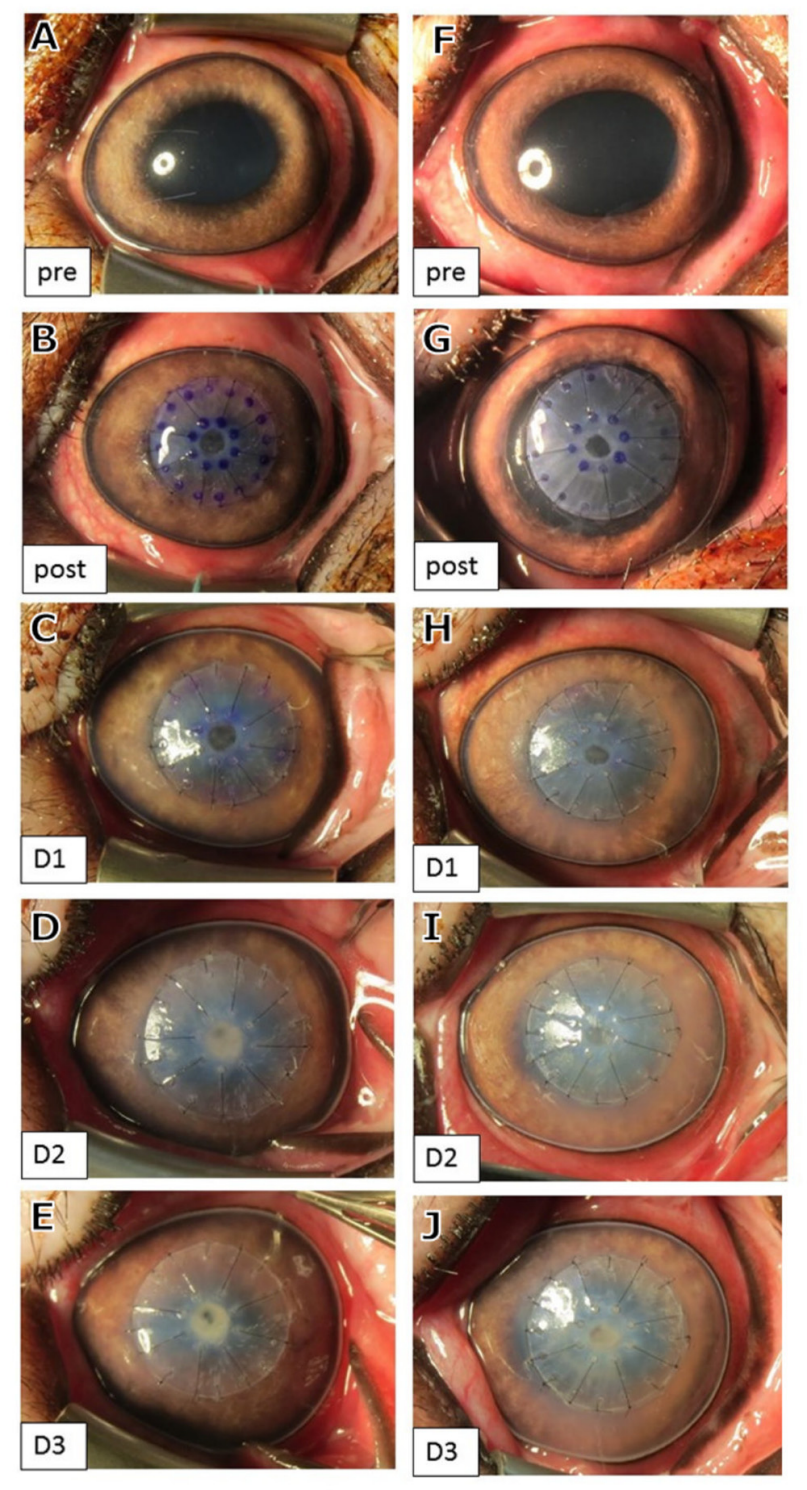
Front view of the corneas patched with type A BioCornea. Two representatives out of eight operated Lany-yu minipigs' corneas were shown for their front views prior (A, F), post (B, G) and 1–3 (C-E, H-J) days after surgeries.

**Fig 2:**
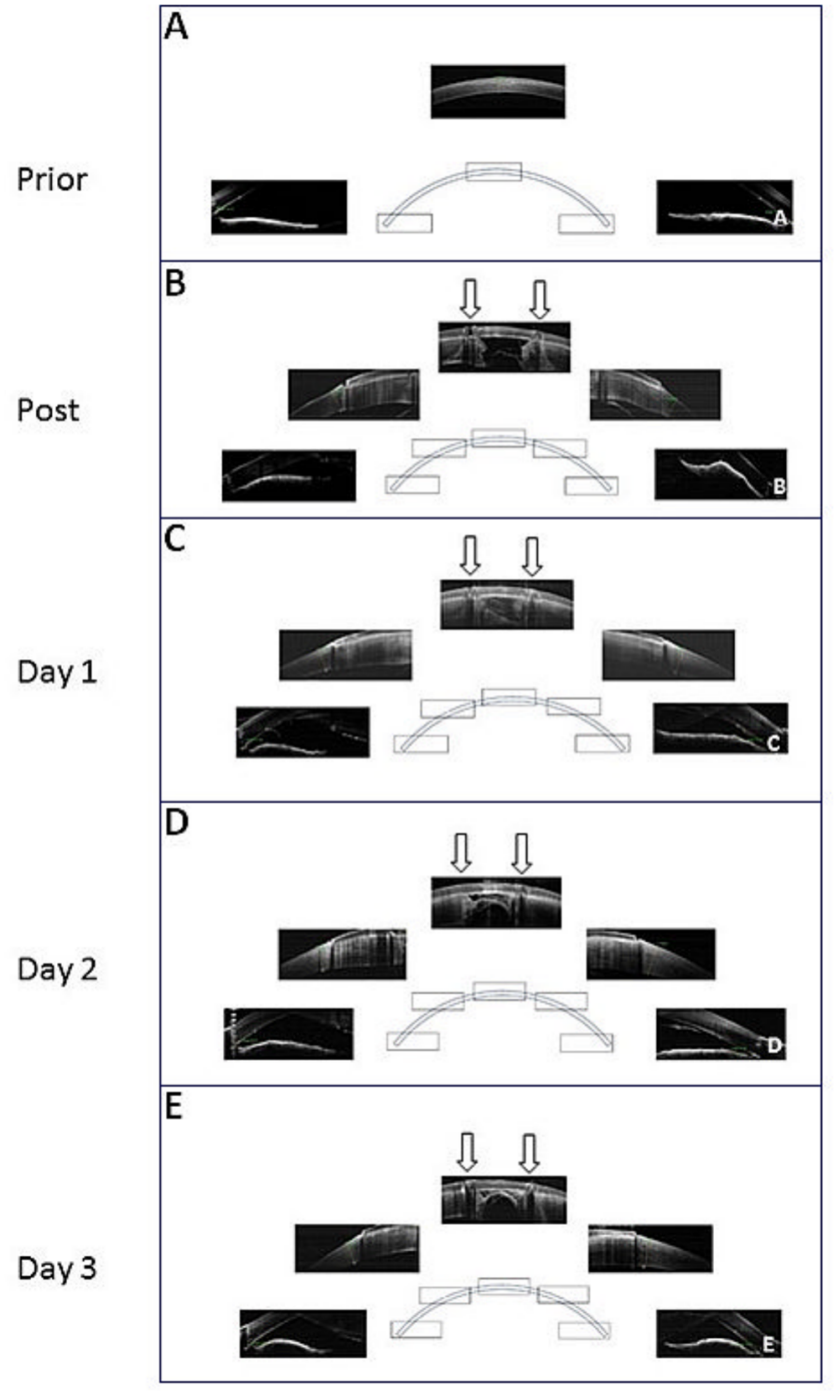
Optical coherence tomography of perforated corneas patched with type A BioCornea. Vertical sections through the central and peripheral cornea are shown. The arrows indicate 2 of the 8 suture holes circulating the peroration, which are located on the inner ring-of-suture-holes.

**Fig 3:**
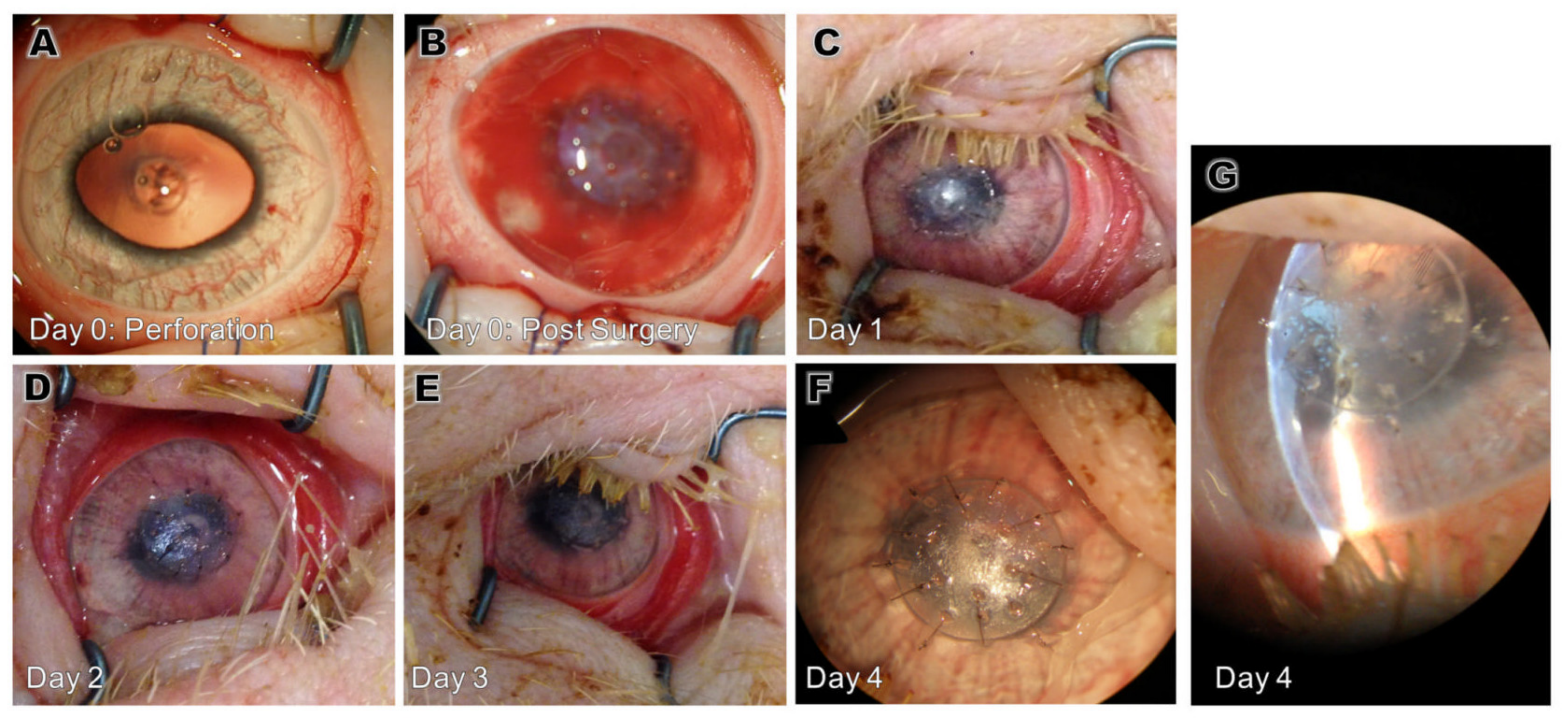
Corneas patched with type B BioCornea in Gottingen mini-pigs. *A*: Perforation. *B*: Post surgery a hyphema occured due to a ruptur of a prominent iris vessel. *C-F*: Photographs from day 1–4. A minor discharge, presumabely fibrin, was seen on the daily examination. *G*: Slit lamp photograph from day 4.

All 8 Lan-Yu mini pigs were succesfully operated and fitted with type A BioCorneas. Photographs of 2 representative operated corneas patched with Type A BioCornea are shown in Fig 1 and shows the state of the eye over three days. Within the period no leakage of aqueous humour through the perforated wounds was observed. The condition of the perforated corneas were found stable without notable acute inflammation and corneal haze. However, moderate swelling were observed from day1 to day 3 after patching (Fig 1C–1E and 1H–1J). To examine the interface between the BioCornea and the corneas, as well as the integrity of anterior chamber, optical coherence tomography (OCT) was used. Prior to trephinations, the integrity of the cornea was confirmed with OCT imaging (Fig 2A). The average central corneal thickness (CT) of Lan-Yu minipigs was 704±22 mm (n = 8), while the anterior chamber angle (ACA) was 32.5±2.0° (n = 8). Post-operative OCT imaging allowed us to examine the sutured BioCornea in vertical sections (Fig 3B). According to the OCT images, the perforations appeared to be tightly sealed by the BioCornea. In the following 3 days after patching, conditions of the operated corneas were found stable (Fig 2C–2E). The average CT and ACA of each patched cornea were 1136±113 mm and 22.9±5.5° on day 1, 1195±92 mm and 19.4±11° on day 2, and 1146±89 mm and 13.7±11° on day 3, respectively. Corneal thickness was significantly thicker throughout the study period compared to pre-surgery (p<0.05). Swelling did not increase after day 1 (p>0.05) indicating that conditions of the patched perforated corneas was stabilized. Anterior chamber angle was significantly smaller on day 1 compared with pre-surgery (p<0.05), but no further shallowing of ACA occurred after day 1 (p>0.05). We conclude that type A BioCornea is capable to seal the 2-mm full-thickness corneal perforation and maintain the integrity of anterior chamber in Lan-yu minipigs.

A smaller and thinner BioCornea, the type B BioCornea, was tested in 3 Gottingen mini-pigs (Fig 3). All pigs underwent surgery succesfully. One pig developed a hyphema during surgery, which resolved spontaneously within 24 hours (Fig 3). Daily assessment of the eyes and the patches showed a deep anterior chamber without signs of infection though mild corneal edema was observed (Table 2). A mild discharge, presumably fibrin was found in all animals.

**Table 2:** Schematic table of average score ± SD of operated mini-pigs. Gottingen minipigs patched by type B BioCornea were followed 4 days after surgeries. The average scores of several standard parameters were documented.

Parameter	Daty 1	Day 2	Day 3	Day 4
Animal welfare	Good (3/3)	Good (3/3)	Good (3/3)	Good (3/3)
Secretion (0–4)	0.67±0.6	1.0±0	2.0±0	2.0±0
Hyperemia (0–4)	3.0±0	2.7±0.6	2.7±0.6	2.3±0.6
Corneal edema (0–4)	0.33±0.6	1.0±0	0.67±0.6	0.33±0.6
Corneal infiltrate	None (3/3)	None (3/3)	None (3/3)	None (3/3)
BioCornea state	In place (3/3)	In place (3/3)	In place (3/3)	In place (3/3)
Leakage	No (3/3/	No (3/3/	No (3/3/	No (3/3/
Anterior chamber depth	Deep (3/3)	Deep (3/3)	Deep (3/3)	Deep (3/3)
Anterior chamber flare	None (3/3)	None (3/3)	None (3/3)	None (3/3)
Iris hyperemia	1.6±2	1.6±2	1.3±2	1.3±2
Pupil	Centered (3/3)	Centered (3/3)	Centered (3/3)	Centered (3/3)

**Fig 4:**
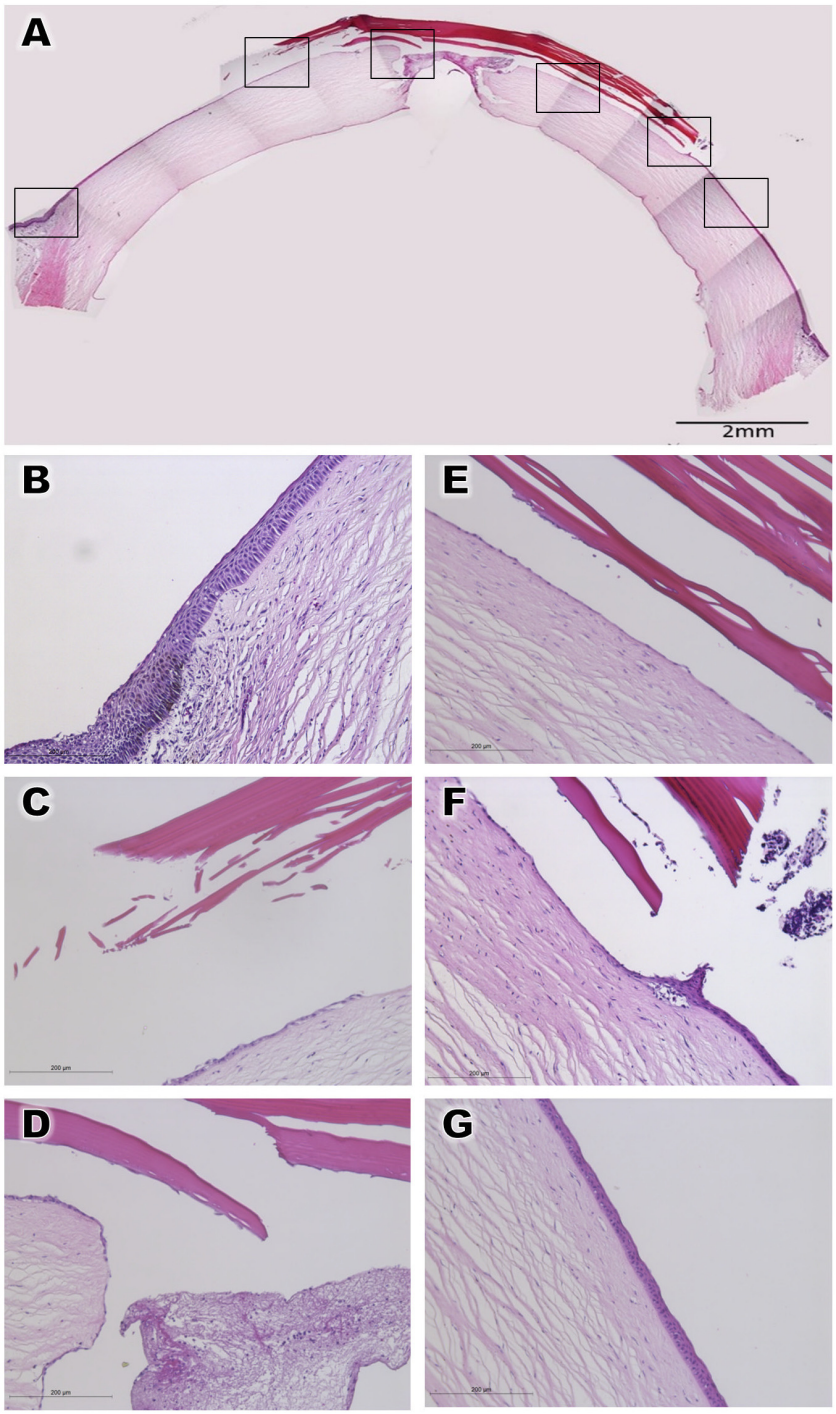
Hematoxylin and eosin staining of perforated cornea patched with BioCornea Type A. *A*: The whole specimen *B-G*: Detailed view of the areas marked by boxes in panel A.

**Fig 5:**
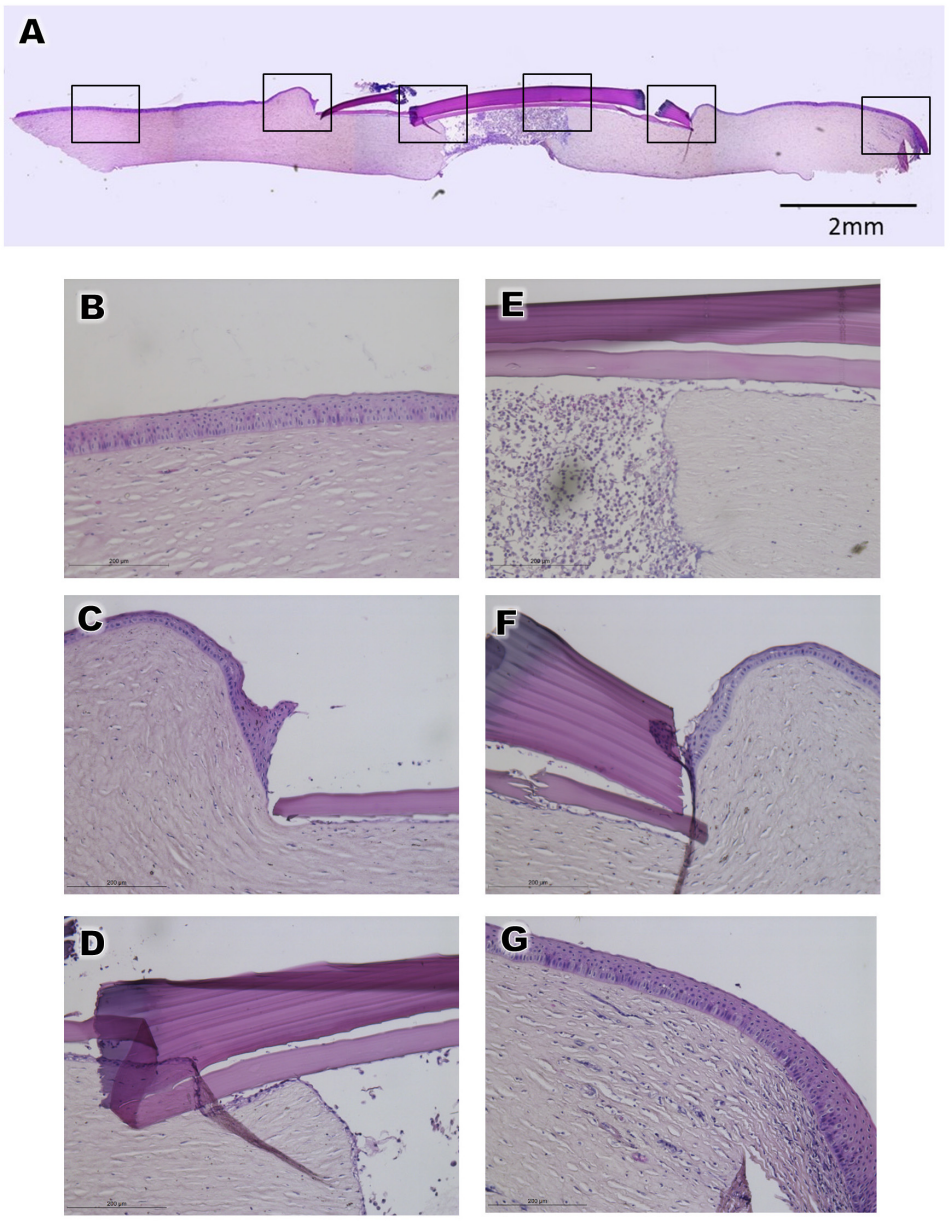
Hematoxylin and eosin staining of perforated cornea patched with BioCornea Type B. *A*: The whole specimen *B-G*: Detailed view of the areas marked by boxes in panel A.

The type B BioCornea was thus also found to be well tolerated and could successfully seal the perforation. Hematoxylin and eosin (HE) staining showed that the perforated corneas patched by either Type A or Type B BioCornea were temporized in 3 and 4 days, respectively (Figs 4 and 5). Moderate swelling was noted in the perforated cornea, and the HE staining clearly showed that the corneal epithelium beneath the placed BioCornea became thinner. No epithelial ingrowth to the penetrating wound was observed, nor severe leucocyte infiltration was noted (Figs 4 and 5).

We concluded that BioCornea is safe to be applied as a corneal patch to seal full-thickness perforation. No adverse events occured in all tested animals and the applied BioCorneas were found well tolerated in all sutured corneas during the study period.

## Discussion

The concept of an artificial cornea was proposed as early as more than 200 years ago [25]. Over the years several attempts have been made to construct cornea prostheses including the Boston Keratoprothesis, AlphaCor, and the osteo-odonto keratoprothesis. These products are however intended as a permanent solution and are most commonly used as a last resort after corneal transplantation failures or in cases deemed unsuitable for transplantation. The use of BioCornea described in this report differs from the artificial corneas mentioned above in that it is intended for acute and short-term use and does therefore complement the products currently available.

In the present study, the ability of BioCornea to close a 2-mm penetrating corneal perforation was evaluated in pig models. The main outcome parameter was if BioCornea could seal the perforation for 3–4 days and was assessed with OCT imaging and handheld slit lamp. The results shown in this report confirm the capability of BioCornea in sealing corneal perforations, with out any adverse events. Histological analysis showed no sign of inflammation.

Two versions of BioCornea with slightly different specifications were tested. After the successful use of the Type-A BioCornea, a refined version, Type-B, was developed and tested. The Type B BioCornea is smaller in diameter and thinner in central thickness compare with Type-A. It requires fewer sutures to completely seal the perforation, therefore it is easier to use in clinical practice and reduces the risk irritation in the eye. Although the animals that received the type B BioCornea were not assessed with advanced OCT imaging, we believed that examination with handheld slit lamp is sufficient for proof of concept.

Clinical cases of traumatic corneal perforations with loss of stromal tissue that need to be repaired with a graft material are quite rare. However, progressions of corneal melting and/or ulcers from different etiology are the more common causes that need immediate surgical repair. Minor leaks can be treated with cyanoacrylate glue, amniotic membrane patch grafts, or Tenon’s membrane auto-grafts, while larger perforations need more firm closure with human donor graft materials. In many countries, a donor cornea can be supplied from a cornea bank within 24 hours, or corneal tissue remnants from another same-day keratoplasty procedure may be available. If human corneal donor tissue is unavailable from such sources, certain synthetic or semi-synthetic patchable material could serve as a transition to a real, permanent keratoplasty procedure. A limitation of this study is that both types of the BioCornea were evaluated in eyes with traumatic perforations but we believe that BioCornea is also suitable for other types of perforations.

Recent trends in the development of artificial corneas move toward the use of materials derived from native sources and materials biologically inspired by native cornea [26]. The most studied material from native sources is decellularized porcine corneal stroma (PCS), while BioCornea is a good example of a material inspired by native cornea [22, 27]. The advantage of decellularized porcine corneal stroma is its great resemblance to human cornea, both in terms of light-transmission and biophysical and chemical properties. However these properties are difficult to maintain during the decellularization processes leading to a PCS [28, 29, 30, 31]. On one hand, it is crucial to remove all the cell debris, DNA contents, and xenogenic antigens to reduce immunogenicity. But on the other hand the transparency and water absorbance ratio are jeopardized by the detergents and chemicals used for PCS decellularization. Thus it’s difficult to obtain a totally non-immunoactive PCS with perfect cornea-like properties in a cost-effective way. This can however be achieved by using fish scale. During the decelluarization and decalcification process, the fish scale becomes more and more “cornea-like” in terms of transparency and tensile strength, while the cell residues are being removed by the strong detergents/chemicals [22]. This results in a transparent articial cornea with very low risk of inducing an immune [23,24]. Whereas the two versions of BioCornea used in this study have not been studied long-term, similar fish-scaled derived artificial corneas have successfully maintained transparency for 6 months. It is therefore likely that the BioCornea can maintain transparency for months, but we emphasize that the intended use is only a few days.

We conclude that BioCornea can be safely used to seal quite large full-thickness corneal perforations for at least 3 days. We recommend the use of type B BioCornea based on the fact that it is easier to handle. BioCornea is a ready-to-use stockable corneal patch that has the potential to become a standardized temporary treatment for corneal perforations, particularly in the medical centers without a cornea bank in close vicinity. Clinical trials are needed to test the device in humans.

## Supporting Information

S1 Fig Photos of all Lany-yu pigs after surgery. (TIF)

S1 Table Raw data from OCT imaging of Lany-yu minipigs after surgery. (XLSX)

## References

[1] Lekskul M, Fracht HU, Cohen EJ, Rapuano CJ, Laibson PR. Nontraumatic corneal perforation. Cornea. 2000;19:313–319 10832690

[2] Hamill MB. Corneal and scleral trauma. Ophthalmol Clin North Am. 2002;15:185–194. 12229235 10.1016/s0896-1549(02)00018-4

[3] Jhanji V, Young AL, Mehta JS, Sharma N, Agarwal T, Vajpayee RB. Management of corneal perforation. Surv Ophthalmol. 2011;56:522–538 10.1016/j.survophthal.2011.06.003 22117886

[4] Michael JG, Hug D, Dowd MD. Management of corneal abrasion in children: a randomized clinical trial. Ann Emerg Med. 2002;40:67–72. 12085075 10.1067/mem.2002.124757

[5] Omobolanle AA, Henrietta N. Pattern of paediatric corneal laceration injuries in the University of Port Harcourt teaching hospital, Rivers state, Nigeria. BMC Res Notes. 2012;5:683 10.1186/1756-0500-5-683 23234255 PMC3546956

[6] Portnoy SL, Insler MS, Kaufman HE. Surgical management of corneal ulceration and perforation. Surv Ophthalmol. 1989;34:47–58. 2678553 10.1016/0039-6257(89)90129-x

[7] Hart A, White S, Conboy P, Quinton D. The management of corneal abrasions in accident and emergency. Injury. 1997;28:527–529. 9616389 10.1016/s0020-1383(97)83472-9

[8] Aslam SA, Sheth HG, Vaughan AJ. Emergency management of corneal injuries. Injury. 2007;38:594–597 16949077 10.1016/j.injury.2006.04.122

[9] Vora GK, Haddadin R, Chodosh J. Management of corneal lacerations and perforations. Int Ophthalmol Clin. 2013;53:1–10 10.1097/IIO.0b013e3182a12c08 24088928

[10] Grau AE, Durán JA. Treatment of a large corneal perforation with a multilayer of amniotic membrane and TachoSil. Cornea. 2012;31:98–100. 10.1097/ICO.0b013e31821f28a2 21963863

[11] Kara S, Arikan S, Ersan I, Taskiran Comez A. Simplified technique for sealing corneal perforations using a fibrin glue-assisted amniotic membrane transplant-plug. Case Rep Ophthalmol Med. 2014:351534 10.1155/2014/351534 25045563 PMC4087251

[12] Kim EC, Jee D, Kim J, Kim MS. Regeneration of cornea long after amniotic membrane grafting to treat corneal perforation. Can J Ophthalmol. 2010;45:9–10. 21179182 10.1139/i10-075

[13] Nubile M, Carpineto P, Lanzini M, Ciancaglini M, Zuppardi E, Mastropasqua L. Multilayer amniotic membrane transplantation for bacterial keratitis with corneal perforation after hyperopic photorefractive keratectomy: case report and literature review. J Cataract Refract Surg. 2007;33:1636–1640 17720083 10.1016/j.jcrs.2007.04.040

[14] Savino G, Colucci D, Giannico MI, Salgarello T. Amniotic membrane transplantation associated with a corneal patch in a paediatric corneal perforation. Acta Ophthalmol. 2010;88:15–16. 10.1111/j.1755-3768.2009.01522.x 19785639

[15] Sharma A, Kaur R, Kumar S, Gupta P, Pandav S, Patnaik B, et al Fibrin glue versus N-butyl-2-cyanoacrylate in corneal perforations. Ophthalmology. 2003;110:291–298. 12578769 10.1016/S0161-6420(02)01558-0

[16] Solomon A, Meller D, Prabhasawat P, John T, Espana EM, Steuhl KP, et al Amniotic membrane grafts for nontraumatic corneal perforations, descemetoceles, and deep ulcers. Ophthalmology. 2002;109:694–703. 11927426 10.1016/s0161-6420(01)01032-6

[17] Rana M, Savant V. A brief review of techniques used to seal corneal perforation using cyanoacrylate tissue adhesive. Cont Lens Anterior Eye. 2013;36:156–158. 10.1016/j.clae.2013.03.006 23623338

[18] Taravella MJ, Chang CD. 2-Octyl cyanoacrylate medical adhesive in treatment of a corneal perforation. Cornea. 2001;20:220–221. 11248835 10.1097/00003226-200103000-00024

[19] Sii F, Lee GA. Fibrin glue in the management of corneal melt. Clin Experiment Ophthalmol. 2005;33:532–534. 16181285 10.1111/j.1442-9071.2005.01076.x

[20] Rijneveld WJ, Wolff R, Völker-Dieben HJ, Pels E. Validation of tissue quality parameters for donor corneas, designated for emergency cases: corneal graft survival. Acta Ophthalmol. 2011;89:734–740. 10.1111/j.1755-3768.2009.01805.x 20039852

[21] Rijneveld WJ, Wolff R, Völker-Dieben HJ, Pels E. Validation of tissue quality parameters for donor corneas designated for emergency use in preservation of the globe. Cornea. 2010;29:128–132. 10.1097/ICO.0b013e3181ac07bc 19966565

[22] Lin CC, Ritch R, Lin SM, Ni MH, Chang YC, Lu YL, et al A new fish scale-derived scaffold for corneal regeneration. Eur Cell Mater. 2010;19:50–57. 20186665

[23] van Essen TH, Lin CC, Hussain AK, Maas S, Lai HJ, Linnartz H, et al A fish scale-derived collagen matrix as artificial cornea in rats: properties and potential. Invest Ophthalmol Vis Sci. 2013;54:3224–3233. 10.1167/iovs.13-11799 23580482

[24] Yuan F, Wang L, Lin CC, Chou CH, Li L. A cornea substitute derived from fish scale: 6-month followup on rabbit model. J Ophthalmol. 2014;914542 10.1155/2014/914542 25089206 PMC4096004

[25] Chirila TV, Hicks CR. The origins of the artificial cornea: Pellier de Quengsy and his contribution to the modern concept of keratoprosthesis. Gesnerus. 1999;56:96–106 10432778

[26] Griffith M, Harkin DG. Recent advances in the design of artificial corneas. Curr Opin Ophthalmol. 2014;25:240–247 24663067 10.1097/ICU.0000000000000049

[27] Ikoma T, Kobayashi H, Tanaka J, Walsh D, Mann S. Microstructure, mechanical, and biomimetic properties of fish scales from Pagrus major. J Struct Biol. 2003; 142:327–333. 12781659 10.1016/s1047-8477(03)00053-4

[28] Du L, Wu X. Development and characterization of a full-thickness acellular porcine cornea matrix for tissue engineering. Artif Organs. 2011;35:691–705. 10.1111/j.1525-1594.2010.01174.x 21501189

[29] Zhou Y, Wu Z, Ge J, Wan P, Li N, Xiang P, et al Development and characterization of acellular porcine corneal matrix using sodium dodecylsulfate. Cornea. 2011;30:73–82 20861730 10.1097/ICO.0b013e3181dc8184

[30] Lynch AP, Ahearne M. Strategies for developing decellularized corneal scaffolds. Exp Eye Res. 2013;108:42–47 10.1016/j.exer.2012.12.012 23287438

[31] Lee W, Miyagawa Y, Long C, Cooper DK, Hara H. A comparison of three methods of decellularization of pig corneas to reduce immunogenicity. Int J Ophthalmol. 2014;7:587–593. 10.3980/j.issn.2222-3959.2014.04.01 25161926 PMC4137190

