# Supplementary figures and images for: Use of Fish Scale-Derived BioCornea to Seal Full-Thickness Corneal Perforations in Pig Models
Source: PLoS One. 2015 Nov 24;10(11):e0143511. doi: 10.1371/journal.pone.0143511 (PMC4657996; doi:10.1371/journal.pone.0143511)

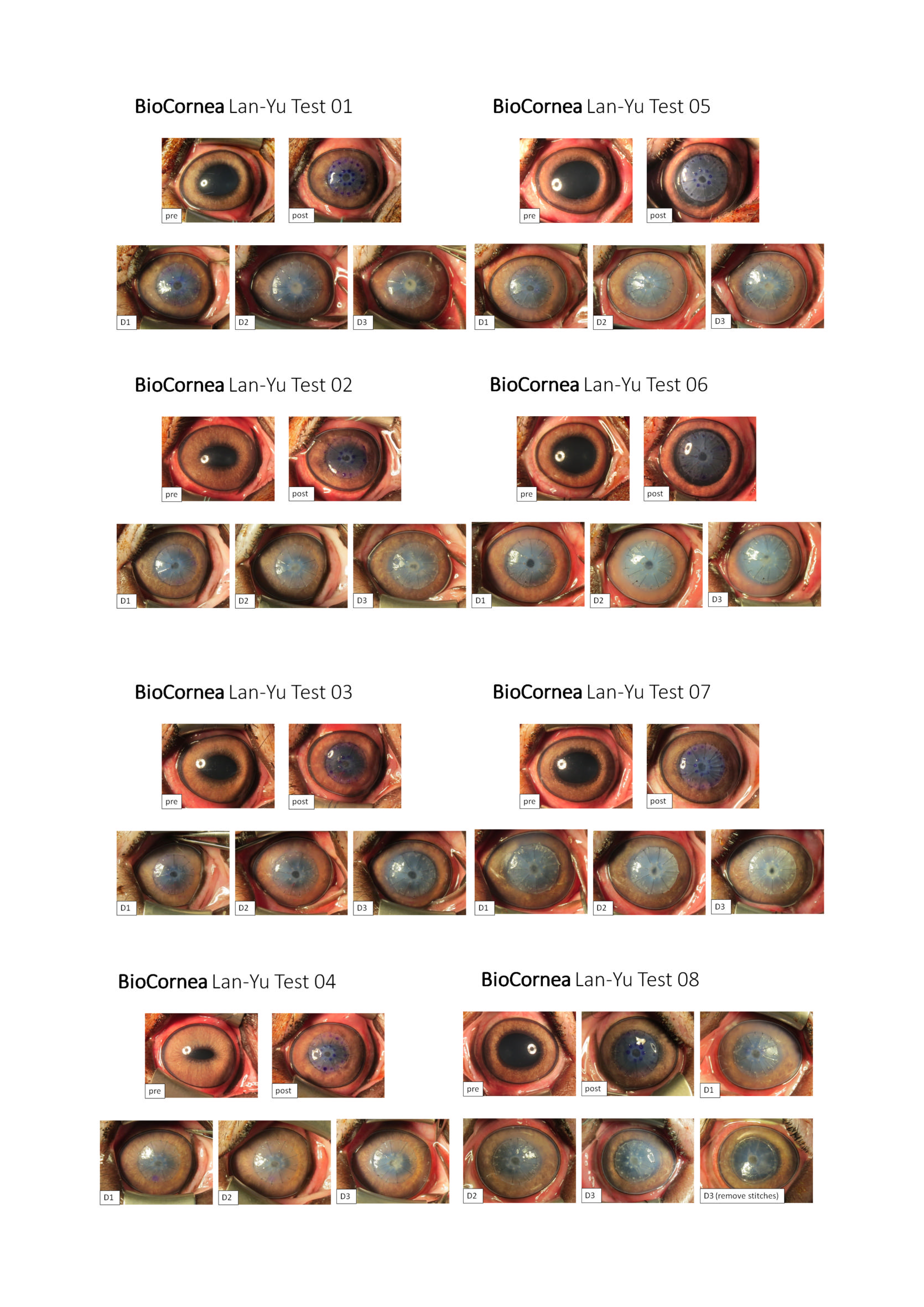

Supplement: S1 Fig — (TIF) [file pone.0143511.s001.tif]
